# Research on Intelligent Warehousing and Logistics Management System of Electronic Market Based on Machine Learning

**DOI:** 10.1155/2022/2076591

**Published:** 2022-03-17

**Authors:** Ruifeng Zhang, Xiaoyan Zhou, Yanfeng Jin, Jing Li

**Affiliations:** Shijiazhuang Posts and Telecommunications Technical College, Shijiazhuang, Hebei 050021, China

## Abstract

This study applies the Internet of things information-aware technology to the process of electronic market warehousing and logistics management, effectively perceives warehouse electronic product logistics information, and improves the real-time perception of electronic product logistics information and the efficiency of electronic product storage logistics management. This study first analyzes the needs of the intelligent electronic market warehouse logistics management system and then builds the IoT architecture of the intelligent warehouse logistics assembly logistics management system for electronic warehouses based on machine learning algorithms, which solves the problems that exist in the current workshop electronic market warehouse logistics management. Then, the principle of RFID technology is introduced. The accuracy of RFID tag estimation is analyzed by the PEPC tag estimation algorithm. It is concluded that the PEPC tag estimation algorithm reduces the tag estimation error and improves the accuracy of tag estimation. Finally, an intelligent warehousing logistics management system based on IoT RFID technology is established. The test results show that the system can meet the requirements of intelligent warehousing function in the electronic market, which will greatly improve the warehousing efficiency of electronic products.

## 1. Introduction

With the rapid development of science and technology, it is necessary to produce a wide variety of electronic products, and almost every 10,000 products are produced every minute and stored in various fields of society to meet the needs of consumers. How to perceive the logistics information of electronic products is an urgent problem for enterprises to solve.

The application of Internet of things RFID technology in the field of warehousing logistics management has always been a hot issue in academic and industrial research. La-or Kovavisaruch et al. [1] applied RFID technology to the management of truck information in the logistics industry. Jiahao Wang et al. [2] studied the related problems of the automatic assembly line of manufacturing based on RFID technology. Song et al. [3] designed and developed an automatic tracking system using RFID technology for the long-term discrete manufacturing supply chain and the difficulty in tracking products during storage, installation, and production inspection. He Wei et al. [4] realized the real-time data acquisition and optimization scheduling management in the discrete manufacturing process by combining multiple information-aware technologies. Tian Meihua et al. [5] used RFID technology to realize the automatic perception of production data in the Haier production line. Chen Xing et al. [6] used RFID technology to realize the perception and transmission of the underlying data of the MES.

Scholars at home and abroad have always focused on the research of static logistics scheduling and dynamic scheduling (also known as intelligent scheduling of storage system) [7–9]. The research scenarios involve automated terminals, production workshops, automated warehouses, logistics distribution centers, etc. The static scheduling method needs to obtain a batch of task information and system status in advance and will not change the scheduling strategy until the task is completed. The emergence of intelligent warehouse logistics dynamic scheduling can make up for the shortcomings brought by static scheduling, but the dynamic scheduling method mentioned in the research of dynamic scheduling method is actually a rescheduling method, which will readjust the scheduling strategy according to the system state [10–14]. However, in complex environments, the calculation and evaluation of intelligent algorithms such as genetic algorithm and particle swarm optimization take a long time and often cannot meet the real-time requirements of the system. The research on the dynamic scheduling of intelligent warehousing logistics by scientific researchers mainly includes two categories. One is to use the knowledge base system to write the optimal scheduling strategies in different states into the knowledge base in advance and select the corresponding scheduling strategies in the knowledge base according to the state in the operation process [15]. However, this method depends on the scale of the knowledge base. If the knowledge base is difficult to cover all possible system states, it will seriously affect the performance of scheduling decision-making. The second is to use the machine learning method to learn the scheduling strategies under different system states offline [16, 17].

In this study, we study the application of IoT information sensing technology in the process of electronic product warehousing and logistics management based on machine learning algorithm through the analysis of product warehousing process in electronic market.

## 2. Demand Analysis of Intelligent Warehousing and Logistics System in Electronic Market Based on Internet of Things

On the basis of the Internet of things, the electronic market intelligent warehousing logistics management system integrates the management and quality control of the supply chain to form complete and orderly information data. Effective measures are taken in a timely manner, so that quality service and faster response speed meet the increasingly personalized needs of customers and then optimize the entire system [18].

### 2.1. Based on the Demand Analysis of Intelligent Warehouse Logistics Management System in the Internet of Things

In the actual warehousing activities, the two functions of storage and distribution are closely connected and even merged together. As for the storage function, there are more distribution functions, depending on the inventory of the goods. The longer the inventory cycle of the goods, the more prominent the storage function. On the contrary, the more prominent the distribution function is, the storage of zero inventory is mainly based on the distribution function. Seasonal stocks and reserve stocks generally correspond to storage-based warehousing, while intermediate conversion inventories generally correspond to storage-based warehousing.

Through the real-time monitoring of the warehouse location, the overall cargo utilization rate and cargo turnover speed are improved, and the detectable grid management of the cargo space further improves the intelligence of warehouse management. Containers and cargo spaces can present corresponding intelligent and physical properties, facilitating unified data management [19].

#### 2.1.1. User Requirements

In the system design, the first step is to design and develop the demand analysis according to the user's needs. The demand analysis is a process of refinement and understanding. The idea used in warehousing logistics management is the management of customers, warehouses, and transportation.

Library management operation principle: the warehouse administrator confirms whether the goods in the warehouse are qualified. If they are qualified, they will be put into the warehouse; otherwise, they will refuse to enter the warehouse. The outbound management is similar to the inbound management: after the user issues the outbound order, the correctness of the item is verified based on the basic data of the material.

#### 2.1.2. Function Module

The intelligent warehouse management system based on Internet of things RFID technology studied in this study mainly includes five modules: basic management, cargo space management, cargo management, warehouse management, and early warning management. Each module also contains several subfunctions. Basic management includes user management, warehouse settings, system settings, and initialization settings. Location management includes shelf management, location management, location hardware matching, and code management. Cargo management includes cargo entry, cargo inquiry modification, cargo specification management, and cargo supplier management. Warehouse management includes functions such as inbound management, outbound management, inventory management, and statistical query; early warning management includes functions such as container gateway, location detector, warning information, and detection and repair information.

### 2.2. Business Analysis of Intelligent Warehouse Logistics Management System Based on Internet of Things

The business of warehouse logistics management system based on Internet of things RFID technology is similar to the traditional browser-based PC service, but it presents new features in the current intelligent warehouse logistics environment. The system fully considers these characteristics in business process analysis, making the design of business processes more suitable for business processing in the new environment [20, 21].

#### 2.2.1. Electronic Market Intelligent Warehousing Logistics System Overall Business Process

**Figure 1:**
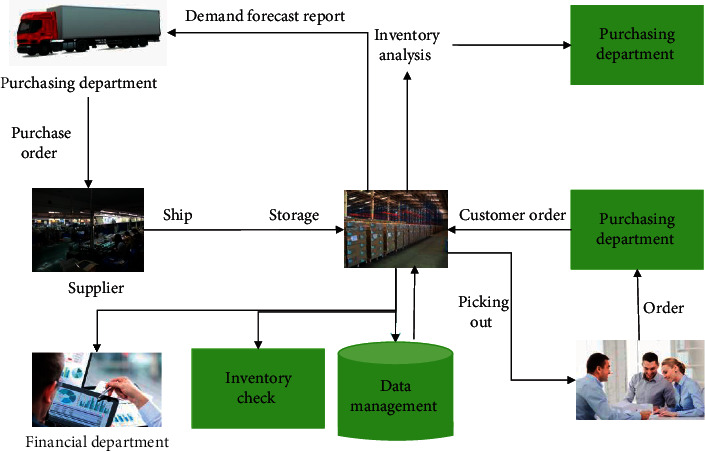
Overall business process of the electronic market logistics management system.

Warehousing intelligent management requires multi-sectoral participation, including procurement department, supplier, receiving warehousing, finance department, inventory counting and other multiparty participation, and multi-process linkage business process. The overall business process of the warehousing and logistics system is shown in Figure 1.

#### 2.2.2. Intelligent Management of Cargo Space

According to certain rules, the location management divides the different container positions in the warehouse into goods of different categories and different specifications. The management of the cargo space is usually distinguished by different codes. It is necessary to make full use of the existing cargo space as much as possible, improve the operational efficiency of the cargo space, and form an efficient and orderly storage operation state with storage and accommodating and accompanying disks.

#### 2.2.3. Dynamic Inventory Business Process

The on-site computer issues a library information command to the mobile disk library to the handheld RFID reading device according to the library plan. The library owner reads the location label and the item label code. If it is found that the two cannot match each other, the user is alerted to the field computer. After the library owner travels once according to the specified route, the library statistic information is sent to the on-site computer. Here, the characteristics of inventory intelligence (recurring intelligent information reminder, periodic reminder of different areas, intelligent reminder of the entire warehouse inventory, etc.) are fully used. Through the unreasonable need to inventory the goods in the warehouse, the quantity of the goods on the book is determined and is sent to the warehouse inventory personnel according to the inventory plan and then the goods corresponding to the difference according to the system feedback of the inventory are adjusted and confirmed.

#### 2.2.4. Warehousing Management Service

The specific process of product warehousing in the electronic market is as follows:

- The warehousing business begins, collecting the basic information of the warehousing, such as the warehousing order information and the goods warehousing details, and transferring the warehousing order to the WMS and generating the warehousing notice, which is received by the operator.
- Data entry container label: the inbound item is encoded with the selected item encoding scheme, the encoded information is written into the electronic label, the paper label is printed, and then the paper label and the electronic label are bonded together to become the goods label.
- Whether the quantity received and the data displayed by the label match is determined. If it matches, it is submitted to the background. The field computer automatically allocates the location and gradually downloads the location number and corresponding item number of each operation to the wireless data terminal. If the data do not match, the goods receipt operation or the feedback data difference is recompleted, and the receiving operation is completed; that is, the receiving business is terminated early.
- The goods are transported by the warehouse staff to the corresponding cargo space. After the checked information is correct, the goods are put into the cargo space (if necessary, the cargo number and data information of the location label can be modified); the real-time storage is collected through the mobile terminal. Information is transmitted to the system to ensure real-time data updates.

#### 2.2.5. Outbound Management Business

The specific process for product delivery in the electronic market is as follows:

- The system center issues an outbound planning instruction.
- The outbound system generates an outbound business order and transmits it to the mobile terminal.
- The staff finds the corresponding location based on the messenger of the mobile terminal.
- The corresponding goods are taken from the cargo position, and the system automatically updates or is in the state.
- The goods are transported to the exit, and the information of the outbound operation is sent back to the on-site computer through the fixed reading and writing device system.
- Then, the seal label is read through the handheld device and is checked.
- Finally, the central database information is updated to end the outbound business process.

#### 2.2.6. Supplier Management Business

The main function of the supplier management business is the supplier's view, addition, modification, deletion, deactivation, etc. The detailed process is as follows:

  ① After the service starts, the system automatically verifies the login information. If the verification fails, the staff prompts the user to reenter the information and then reverify, and until the verification is successful, the supplier basic information is loaded.
  ②The supplier information is entered, such as company name, number, and contact. By matching the common information of the supplier, it is judged whether the information exists, and if it does not exist, the relevant processing is performed; in the case where the information exists, the supplier information is viewed, and the related goods are associated.
  ③ The staff and the supplier negotiate with each other, or add, modify, or delete the cargo interaction information. In special cases, the supplier cooperation may be deactivated.

## 3. The Overall Structure of the Intelligent Electronic Market Warehousing Logistics Management System Based on the Internet of Things

The characteristics of intelligent warehousing logistics based on machine learning are reflected in two aspects: on the one hand, it is intelligent. Intelligent warehousing logistics adopts a large number of relevant technologies perceived by the Internet of things (RFID tags and sensors). Through the intelligent warehousing logistics system, the current state of warehouse materials can be seen in real time, and the previous movement track of this material can be queried. In case of abnormal conditions of materials in the process of warehouse management, the intelligent warehouse logistics system will actively judge the information of abnormal conditions and send an alarm. The other is automation. Intelligent warehousing and logistics based on machine learning connect scheduling and data processing. After obtaining the information, the system integrates and analyzes the data according to the set logic and gives the optimal solution based on a large amount of historical data and intelligent analysis modeling, and through systematic analysis, enterprise managers can timely understand the current state of warehousing logistics and make correct production decisions based on the system, to make the increasingly rich personalized needs of warehousing more flexible response. Compared with traditional warehousing, intelligent warehousing logistics has many outstanding advantages.

**Figure 2:**
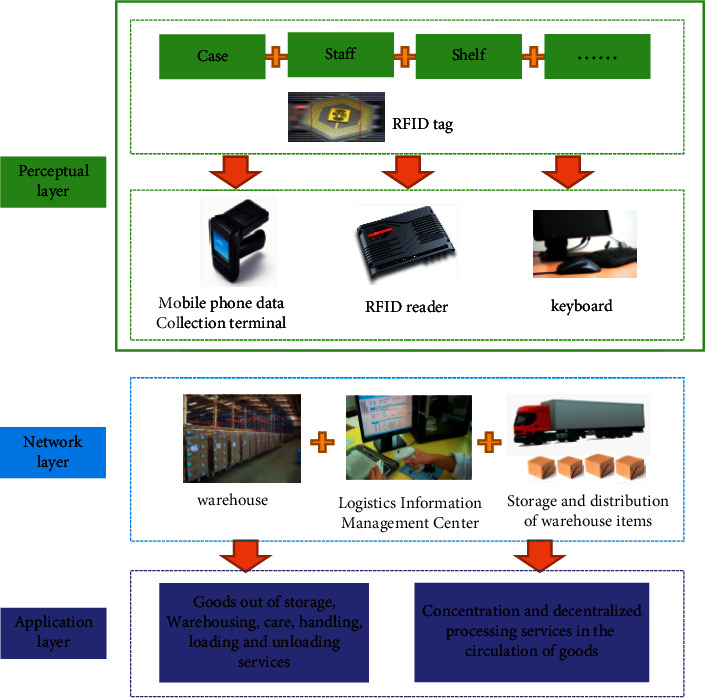
IoT architecture of warehouse logistics management system.

This study applies the Internet of things technology to the process of warehouse logistics management. By constructing the Internet of things architecture of the electronic market warehouse logistics management system, it solves the problems existing in the current electronic market logistics management and meets the application requirements of the electronic market. The IoT architecture of the electronic market warehousing logistics management system is shown in Figure 2. It utilizes the identification of RFID and the perception layer composed of multimode sensing, the network layer of multimode transmission, and the application layer containing core business functions [22–24].

### 3.1. Perception Layer

The perception layer of the electronic market warehousing logistics management system mainly uses the IoT-related information-aware technology to perceive the logistics information generated in the electronic warehouse warehousing logistics management process in real time. In the sensing layer of the electronic market warehousing logistics management system, this study uses the advantages of RFID technology to detect the electronic warehouse warehousing and logistics information in real time by means of RFID identification and multimode perception [25].

According to the storage needs of electronic products purchased from suppliers, after consultation with the customer, electronic products are shipped to the customer. To sense the information of the warehouse electronic product logistics in real time, a fixed RFID reader and a handheld multifunction data collection terminal are arranged in the electronic product warehouse to collect real-time location information of each station in the electronic product. At the same time, a fixed RFID reader and a wireless barcode scanner are arranged at the entrance and exit of the warehouse, and the warehouse administrator is assigned a handheld multifunctional data collection terminal and a computer to collect logistics information of various electronic products in real time.

### 3.2. System Network Layer

The RFID reader, the handheld multifunction data collection terminal, and the barcode scanner are interconnected by means of a field bus or a local area network and organized into a data-aware network, and the electronic product logistics information is stored in real time during the electronic product logistics management process. In the warehouse server, it provides data support for the development and implementation of the electronic market warehouse logistics management system. The warehouse entry port server can transmit information to other departments within the enterprise through the local area network and the intranet and realize information sharing.

### 3.3. Application Layer

Based on the Java EE technology architecture, the electronic market warehouse logistics management system [26] is developed. It is applied to the storage and distribution of items in the electronic market warehousing logistics service. The storage of goods in the electronic market is to provide real-time monitoring functions in the storage environment of goods and in the process of storing goods, such as arrival inspection, warehousing, delivery, care, handling, loading, and unloading. The distribution is a function of providing real-time monitoring in the concentration and dispersion of electronic products on the circulation of goods.

## 4. RFID Tag Estimation Based on PEPC Tag Estimation Algorithm

### 4.1. RFID Technology

**Figure 3:**
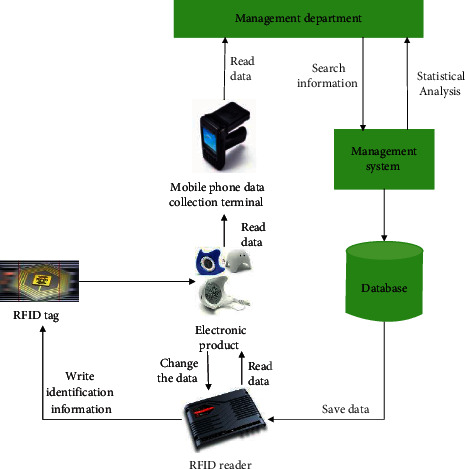
RFID schematic.

RFID [27] technology has become a versatile technology in tag identification and short-range communication and is currently used in many fields. The RFID intelligent electronic market warehousing logistics management system introduces RFID technology into the existing electronic market warehousing logistics management. It performs automated data collection on data of various operations such as inspection, warehousing, delivery, transfer, shifting, and inventory of electronic product warehouses. It ensures the speed and accuracy of data input in all aspects of warehouse management, ensures that the company can accurately grasp the real data of the inventory in a timely and accurate manner, and reasonably maintain and control the enterprise inventory. Through the scientific coding, it is also convenient to manage the batch and shelf life of electronic products. Using the system management function of the system, it is possible to grasp the current location of all the inventory materials in time, which is conducive to improving production efficiency and reducing operating costs. For electronic companies, RFID smart storage systems do not require continuous investment and do not require the purchase of large numbers of tags, making the cost much lower than traditional RFID applications. The RFID principle is shown in Figure 3.

### 4.2. Principle of PEPC Label Estimation Algorithm

The PEPC standard algorithm is used to improve the accuracy of label estimation and adopts the method of pre-estimation and post-checking.

Firstly, the total number of collision tags generated after one round of inquiry is estimated using the tag estimation prediction coefficient, that is, the number of tags to be identified in the next round of reading; when the next round of reading is performed, the number of tags to be identified in the current round is calculated using the collision time slot ratio, that is, the number of collision tags generated in the previous round.

Then, through the evaluation of the next round of label numbers, the evaluation of the previous round of label numbers is compared. According to the comparison result, the prediction coefficient of the previous label estimation is continuously corrected, and the estimation error is reduced to improve the algorithm accuracy.

#### 4.2.1. Frame Length Setting of PEPC Label Estimation Algorithm

The throughput rate is different for different frame lengths. The range of the frame length can be determined according to the following formula:

(1) $$\begin{array}{c} S=\frac{E_{success}}{L}=\frac{n}{L}{\left(1-\frac{1}{L}\right)}^{n-1}. \end{array}$$

The throughput rate under different frame lengths is shown in Figure 3.

**Figure 4:**
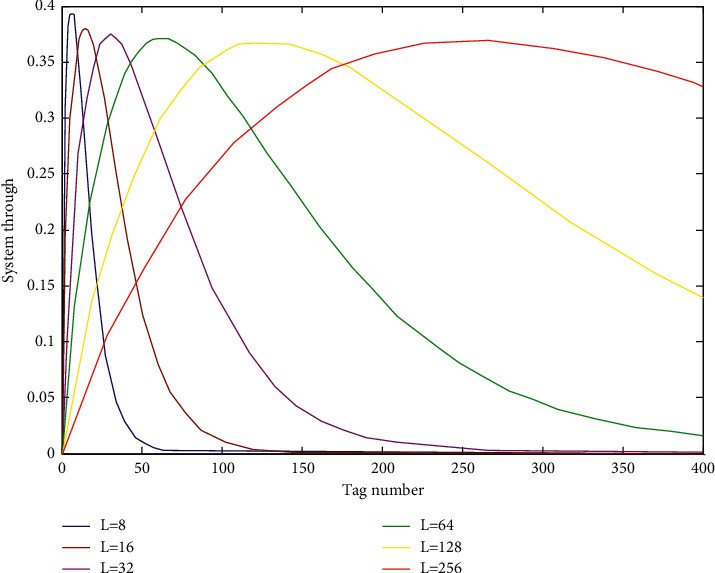
Throughput rate at different frame lengths.

As can be seen from Figure 4, the throughput of the system has its own trend under different frame lengths. Under the two adjacent frame lengths, the system throughput curve has an intersection point. The curve between the two intersection points indicates that when the number of labels is in a certain range, there will be a unique corresponding frame length. The system throughput rate reached a high level. Therefore, this feature satisfies the requirement that the frame length varies dynamically with the tag. These intersection points satisfy the following formula:

(2) $$\begin{array}{c} S\left(L_{1},n\right)=S\left(L_{2},n\right). \end{array}$$

By calculating formula (2), the value of the number of labels in the coordinates of the corresponding intersection can be obtained by the following formula:

(3) $$\begin{array}{c} n=\left[\log_{\left(1-1/L_{2}\right)/\left(1-1/L_{1}\right)}\left(L_{2}/L_{1}\right)+1\right]. \end{array}$$

Therefore, when the number of tags to be identified changes dynamically, a corresponding identification frame length is obtained, as shown in Table 1.

#### 4.2.2. The Flow of the PEPC Label Estimation Algorithm

The flow of the PEPC label estimation algorithm is as follows:

(1) The reader initiates an *i*(*i*=1,2…) query to the tag within its range. After the round of the query, the reader estimates the number of tags that have not been successfully identified, and the step is “pre-estimation.” The number of remaining labels in the round is calculated by the prediction coefficient estimated by the label. Among them, the first round of the query can use the initial frame length and the initial prediction coefficient. The number of unsuccessfully identified tags is as shown as follows:
  (4) $$\begin{array}{c} N_{\text{unread}}=K\cdot C_{\text{collision}}\left(i\right), \end{array}$$
   where *K* is the prediction coefficient, which is the average number of tags in the CS; *C*_collision_(*i*) indicates the number of CSs after the end of the ith query; and *N*_unread_ indicates the number of unrecognized tags after the end of the ith query obtained by “pre-estimation.”
(2) After the ith query ends, the reader immediately sends the *i* + 1th query. After the reading is completed, the collision probability of the time slot is calculated using the frame length and the CS number, and then, the labels of the current round of the query are estimated. The number is the “post check.” See the following formulas:
  (5) $$\begin{array}{c} C_{ratio}^{\left(i+1\right)}=1-{\left(1-\frac{1}{L^{\left(i+1\right)}}\right)}^{N_{final}}-\frac{1}{L^{\left(i+1\right)}}\cdot \left(\left(1-{\frac{1}{L^{\left(i+1\right)}}}^{N_{final-1}}\right)\right), \end{array}$$
  (6) $$\begin{array}{c} N_{final}=F_{trans}\left(C_{ratio}^{\left(i+1\right)},L^{\left(i+1\right)}\right), \end{array}$$

where *C*_*ratio*_^(*i*+1)^ is the ratio of collision time slots generated by the tag during the *i*+1th round of reading;

 
*L*^(*i*+1)^ is the frame length of the label during the *i*+1th round of reading;
 
*N*_*final*_ is the number of tags to be identified when the tag is queried in the *i*+1th round; and
 
*F*_*trans*_ is “Beyond equations” with unknown *N*_*final*_. (Note: the transcendental equation generally has no analytical solution, but only numerical solutions or approximate solutions. The approximate solution is often obtained by the Newton tangent method, power series solution, etc., or using the program compiled by MATLAB.)

Through the “pre-estimation” and “post-validation,” the error *e* between the two can be obtained, as shown as follows:

(7) $$\begin{array}{c} e=N_{final-}N_{unread}. \end{array}$$

The square of the error is represented as follows:

(8) $$\begin{array}{c} e^{2}={\left(N_{final-}N_{unread}\right)}^{2}\\ ={\left(\left(F_{trans}\left(C_{ratio}^{\left(i+1\right)}\right.,L^{\left(i+1\right)}\right)-K\cdot C_{collision}\left(i\right)\right)}^{2}. \end{array}$$

Let ∂*e*^2^/∂*K*=0, and the following formulas are obtained:

(9) $$\begin{array}{c} 2\left(F_{trans}\left(C_{ratio}^{\left(i+1\right)},L^{\left(i+1\right)}\right)-K\cdot C_{collision}\left(i\right)\right)\cdot C_{collision}\left(i\right)=0, \end{array}$$

which is

(10) $$\begin{array}{c} K=\frac{F_{trans}\left(C_{ratio}^{\left(i+1\right)},L^{\left(i+1\right)}\right)}{C_{collision}\left(i\right)}. \end{array}$$

It can be seen from formula (10) that the value of K minimizes the value of *e*, and the prediction coefficient of the reader is variable during each round of reading. It is necessary to continuously adjust the prediction coefficient and reduce the label estimation error. Similarly, the K value can be as shown as follows:

(11) $$\begin{array}{c} K=\frac{F_{trans}\left(C_{ratio}^{\left(i\right)},L^{\left(i\right)}\right)}{C_{collision}\left(i-1\right)}. \end{array}$$

Bringing formulas (11) into (4), after the reader reads the ith wheel, the number of unrecognized tags is as shown as follows:

(12) $$\begin{array}{c} N_{unread}=\frac{F_{trans}\left(C_{ratio}^{\left(i\right)},L^{\left(i\right)}\right)}{C_{collision}\left(i-1\right)}\cdot C_{collision}\left(i\right). \end{array}$$

### 4.3. Machine Learning-Based Algorithm Simulation and Analysis

#### 4.3.1. Analysis of Algorithm under Fixed Frame Length

**Figure 5:**
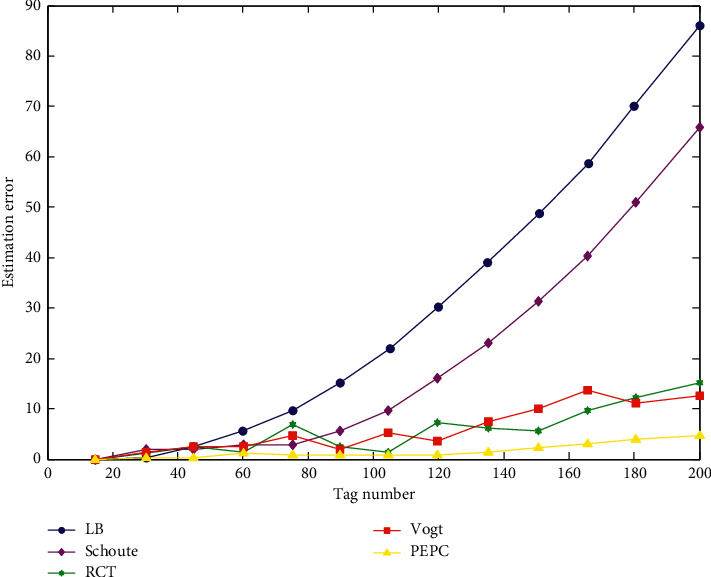
Comparison of error in fixed frame length.

*(1) Simulation Analysis of Tag Estimation Error*. In this experiment, in the MATLAB simulation environment, the number of repeated experiments is 200, and the fixed frame length is 64 bytes. The labels used in the experiment are all generated by random functions. The number of tags to be tested ranges from 0 to 200. The simulation results of the error comparison under the fixed frame length are shown in Figure 5.

It can be seen from the simulation result of Figure 5 that when the number of tags is less than 80 when the fixed frame length is 64 bytes, the error amount of the LB algorithm [28] and the Schoute algorithm is controlled within 10. As the number of tags gradually increases to 200, the error in the estimated value of the tag is also increasing, and the number of errors reaches nearly 90. With such a large error, these two algorithms are only suitable for environments with a small number of tags. Once the number of tags is too large, these two methods will no longer apply. The RCT algorithm and the Vogt algorithm are significantly lower in error quantity than the LB algorithm and the Schoute algorithm. In particular, when the number of labels is less than 90, the error quantity of both algorithms is controlled within 7 and the algorithm is relatively stable. As the number of tags increases, the number of errors in the two algorithms approaches nearly 20. For the PEPC algorithm, the number of errors is significantly better than the existing label estimation algorithm. The number of labels is less than 120, especially the number of errors that does not exceed 1. The PEPC algorithm can basically achieve an accurate estimation of tags. However, as the number of tags increases, the number of errors increases. When the number of tags reaches 200, the number of errors is about 5.

**Figure 6:**
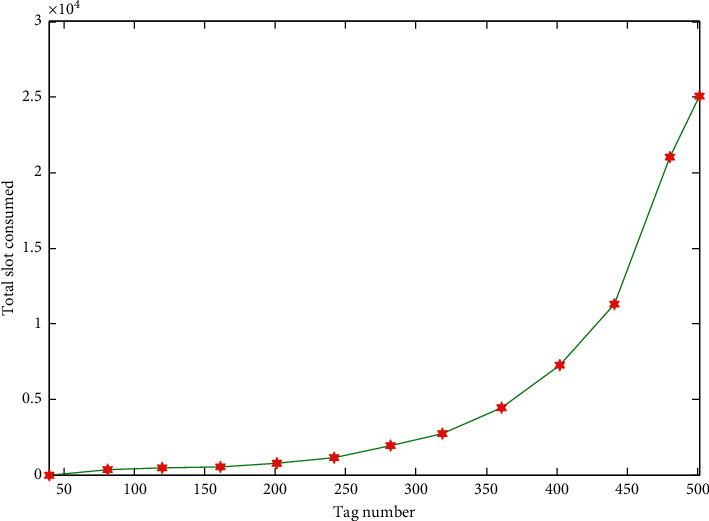
Total number of time slots consumed by the algorithm under fixed frame length.

*(2) Simulation Analysis of the Total Number of Time Slots Consumed by the Algorithm*. In this experiment, the experiment was repeated 200 times in the MATLAB simulation environment, and the fixed value of the frame length was 64 bytes. The labels used in the experiment were all generated by a random function, and the number of tags to be tested ranged from 0 to 500. The simulation results of the total number of time slots consumed by the algorithm under its fixed frame length are shown in Figure 6.

Figure 6 is an analysis of the total number of time slots consumed by the algorithm. When the frame length is fixed, the tag estimation algorithm does not function to dynamically adjust the frame length. Therefore, in a normal environment, the tag responds to the read command of the reader and randomly selects to communicate in the 64 time slots. After each round of identification, the collision label will be randomly selected in the next 64 time slots, and communication will be performed again. Each round of reading will consume 64 time slots. It can be seen from the trend of Figure 6 that when the number of tags is small, the number of time slots consumed by the system increases slowly. Once the number of tags increases, the total number of time slots consumed in the graph increases exponentially. This is because 64 time slots in one round of reading have been unable to efficiently identify the tag. The larger the number of labels, the almost every collision in each round of reading will collide. After the end of the round, a large number of unidentified tags are transferred to the next round, and the vicious state is continued, so that the rate of correct reading of the tags is slow, and a large number of time slots are consumed. When the number of tags reaches 500, the number of time slots consumed when all tags are read is up to 25,000. Therefore, it can be seen from the above analysis that it is very important to dynamically divide the frame length.

#### 4.3.2. Analysis of Algorithm under Dynamic Condition of Frame Length

When the frame length is constant, the total time slot consumption of each tag estimation algorithm is large, and the system work efficiency is low. Therefore, the dynamic frame length is introduced into each algorithm, and the original tag estimation algorithm and the PEPC algorithm designed in this study are simulated and analyzed.

(1) *Simulation Analysis of Tag Estimation Error*. The simulation parameters of this experiment in the MATLAB simulation environment are as follows: the experiment is repeated 200 times, the labels used in the experiment are all generated by the random function, and the number of tags to be tested ranges from 0 to 200. According to the results obtained in Table 1, the value of the frame length dynamically changes with the number of tags, and the simulation data select an appropriate frame length according to the number of collision tags generated per round. In the next round of identification, the reader sends it to all unidentified tags. The simulation results of the error comparison under dynamic frame length are shown in Figure 6.

**Figure 7:**
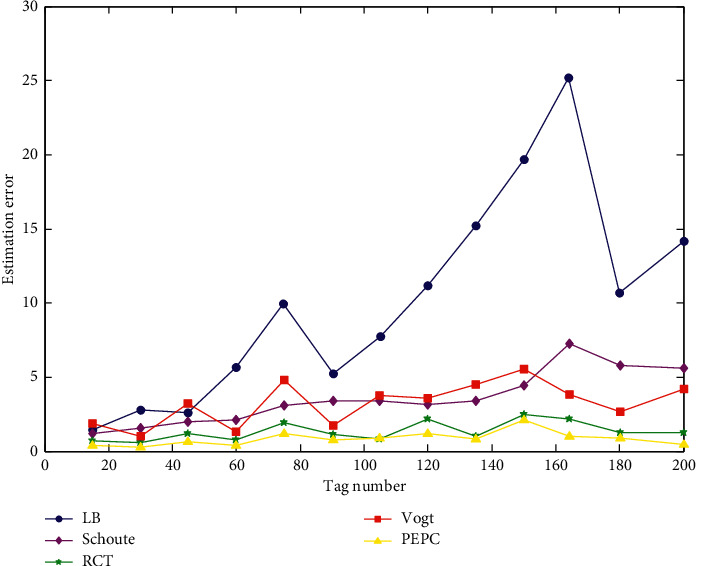
Comparison of error in dynamic frame length.

It can be seen from Figure 7 that after the frame length is dynamically adjusted in the tag identification process, the error amount of all algorithms is significantly reduced. The LB algorithm is one of the most error-prone algorithms, and the algorithm results are unstable and have great volatility. The number of errors between the Schoute algorithm and the Vogt algorithm fluctuates around 5 [29, 30], and the algorithm stability is also poor. The error curves of the RCT algorithm and the PEPC algorithm are less fluctuating, and the error amount is not much different. The RCT algorithm can control the number of errors below 2, and the PEPC algorithm can control the number of errors below 1, closer to the true number of unrecognized tags.

**Figure 8:**
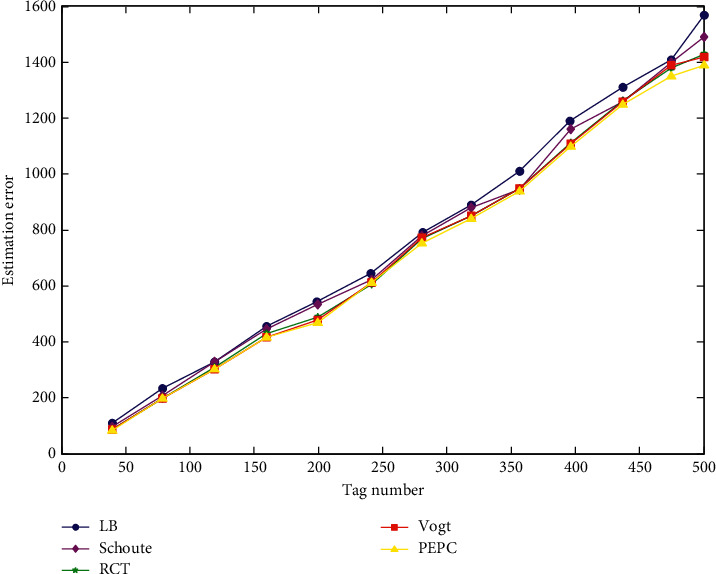
Total number of time slots consumed by the algorithm under the dynamic frame length.

*(2) Simulation Analysis of the Total Number of Time Slots Consumed by the Algorithm*. In this experiment, the number of repeated experiments in the MATLAB simulation environment is 200. The value of the frame length changes dynamically with the number of tags. The tags used in the experiment are all generated by a random function. The number of tags to be tested ranges from 0 to 500. The simulation results of the total number of time slots consumed by the algorithm under its fixed frame length are shown in Figure 8.

Figure 8 is a total number of time slots consumed after all tags have been identified using various tag estimation algorithms. When 500 tags are identified, the LB algorithm consumes the highest total number of time slots and consumes nearly 1600 time slots. The PEPC algorithm is the lowest of the total number of time slots consumed by these five algorithms and consumes nearly 1400 time slots. According to the above simulation results, the PEPC algorithm reduces the label estimation error and reduces the total number of time slots consumed by the tag reading, which can greatly improve the system efficiency.

## 5. Testing of RFID Technology in the Intelligent Warehouse Logistics Management System in the Electronic Market

The electronic market intelligent warehousing logistics management system based on IoT RFID technology designed in this study has tested the function and performance of the system after functional design, business process design, and architecture design.

**Figure 9:**
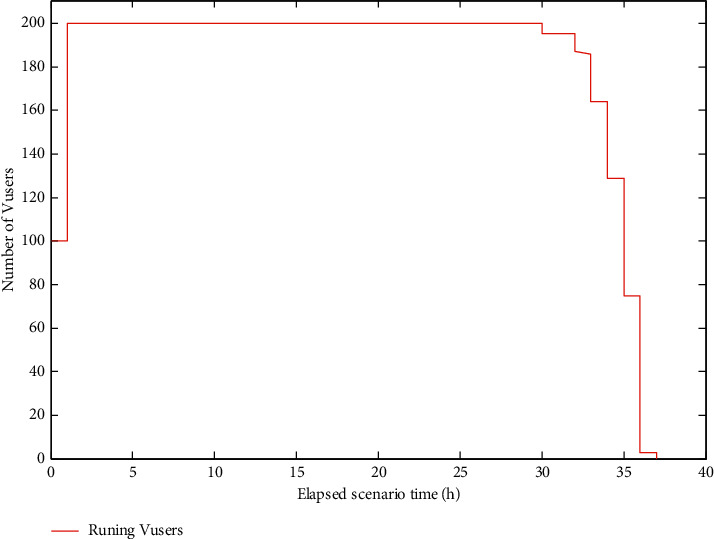
Number of concurrent operations is 200.

In the process of system implementation, the core business of the system is tested according to the test requirements of the user on the performance of the system. The test shows that the execution time of each business of the textbook management system of the university meets the needs of users. The system load runner [31] tests load balancing and monitors the CPU, memory, and traffic resources of the specified application under the use of the machine. The simultaneous execution of 200 users online is shown in Figure 9.

**Figure 10:**
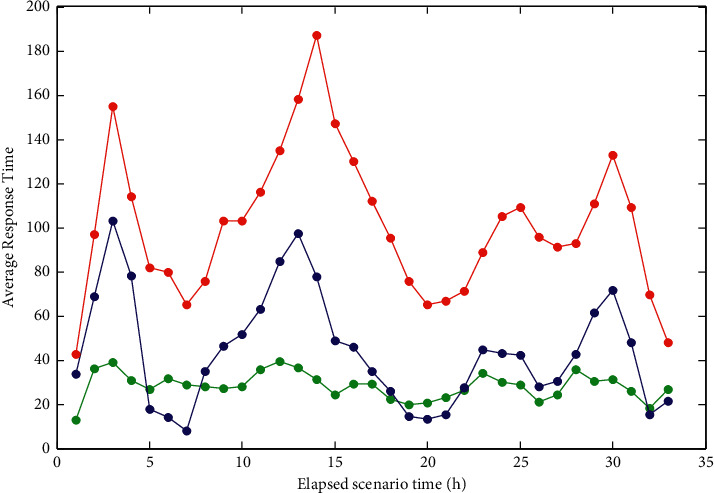
Average transaction response time graph.

The average transaction response time graph is mainly composed of the average transaction response time and the “Transaction Summary” in the result summary, as shown in Figure 10.

**Figure 11:**
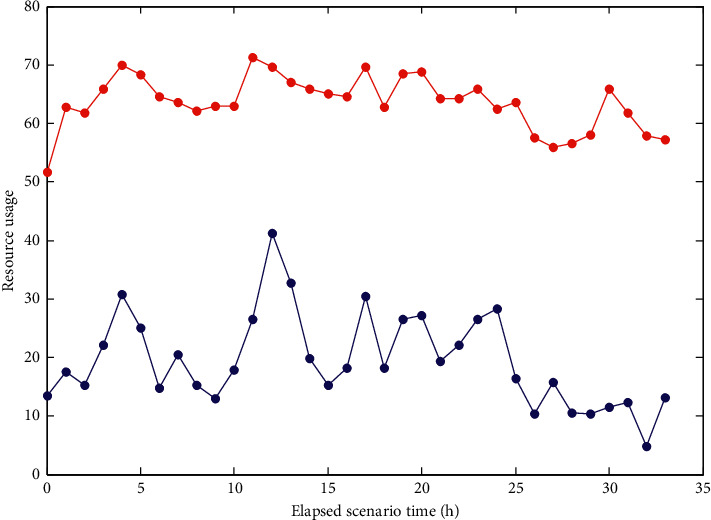
System resource occupancy map.

During the use of the system, the system needs to monitor the resources occupied by the system. This test monitors the CPU and memory usage of the test server and the processor queue, as shown in Figure 11.

As can be seen from Figure 11, the system memory and CPU usage are relatively smooth, the memory usage is about 130 MB, and the CPU usage is over 70%. After the whole chain management of e-commerce warehousing and logistics based on the Internet of things, the following effects have been produced: first, efficiency is improved. RFID technology is used to automatically identify in the process of warehousing, outbound, and inventory, to improve the efficiency of warehouse core business management. Second, manpower is reduced. Compared with the traditional way of recording, scanning, inventory, and other manual actions, it can reduce personnel demand and save labor cost. Third, costs are saved. Resource utilization is increased, operational complexity is reduced, customer response speed is improved, and overall operating costs are reduced.

In the future, there will be more than tens of billions of devices and machines interconnected. Once connected, they must be secure enough to withstand direct intrusion at the hardware level. Because these devices, machines, systems, and networks are becoming more aware of the environment, they must adapt to their environment and needs and must have greater programmability and software definition. In addition, they must be scalable, as more and more features are not only virtualized, but also efficiently mapped to shared computing resources. These systems and networks must also meet the growing needs of end users and real-time scenarios that require immediate low latency response. Behind the scenes, however, they must also process exponentially increasing amounts of data and packets through more advanced algorithms while minimizing power consumption. It must also be highly differentiated; otherwise, it will fail in an increasingly competitive low-cost global market. This can only be achieved by combining software intelligence with hardware-optimized, “arbitrary” connections. The technical indicators to be achieved by the system are shown in Table 2.

## 6. Conclusions

As the core part of manufacturing enterprises, intelligent warehouse management needs to seek a breakthrough and realize lean reform for logistics management. In this study, MATLAB simulation software is used to simulate the existing label estimation algorithm and PEPC algorithm. Taking the simulation of slave error and total slot consumption as the starting point, the fixed frame length and dynamically changing frame length are discussed. The simulation results show that the PEPC tag estimation algorithm reduces the tag estimation error and improves the accuracy of tag estimation. Finally, it expounds on the implementation of electronic products intelligent warehouse logistics management system on the Internet of things environment and sets up the test environment of the use environment. The test results show that the electronic market intelligent warehouse logistics management system implemented by the system provides convenient logistics management services for warehouse managers and improves the information level and core competitiveness of warehousing enterprises.

In addition, the electronic market intelligent warehouse management system provides information guidance for the upstream and downstream of the supply chain, reduces operation links, and improves resource utilization, so that customers can get corresponding products and services as soon as possible. It is necessary to strengthen system integration, that is, gathering basic resources, electronic information, decision-making, and other elements together to improve the comprehensiveness of the logistics system, so that it can reduce the waste of resources, promote the optimal allocation of each kind of resources, and further promote the realization of the purpose of maximizing enterprise benefits.

## Figures and Tables

**Table 1:** Relationship between frame length and number of labels.

Number of tags to be identified	Identify the frame length (byte)	Number of tags to be identified	Identify the frame length (byte)
1 ≤ *n* ≤ 11	8	12 ≤ *n* ≤ 22	16
23 ≤ *n* ≤ 43	32	45 ≤ *n* ≤ 89	64
90 ≤ *n* ≤ 177	128	*n* ≥ 178	256

**Table 2:** Technical indicators to be achieved by the project.

Metrics	Ranges
Business processing time (ms)	<1800
Business concurrent processing quantity	4350
Business processing average response time (ms)	215
Maximum processing wait time (ms)	175
Mean time between failures (hours)	23850

## Data Availability

The data used to support the findings of this study are available from the corresponding author upon request.
